# The Evolution of the Epidemiology of Facial Fractures Before, During and After the COVID-19 Pandemic

**DOI:** 10.1007/s12663-024-02153-0

**Published:** 2024-03-24

**Authors:** Marzia Petrocelli, Federica Ruggiero, Liliana Feraboli, Davide Allegri, Sebastiano Cutrupi, Anna Maria Baietti, Giovanni Salzano, Gianmarco Prucher, Pietro Maremonti, Luigi Angelo Vaira

**Affiliations:** 1 Oral and Maxillo-Facial Unit, AUSL Bologna Bellaria-Maggiore Hospital, Bologna, Italy; 2 Department of Clinical Governance and Quality, AUSL Bologna Bellaria-Maggiore Hospital, Bologna, Italy; 3 Dentistry Unit, AUSL Bologna Bellaria-Maggiore Hospital, Bologna, Italy; 4 Maxillo-Facial Surgery Unit, University Hospital of Naples “Federico II”, Naples, Italy; 5 Maxillofacial Surgery Operative Unit, Department of Medical, Surgical and Experimental Sciences, University of Sassari Hospital, University of Sassari, Viale San Pietro 43B, 07100 Sassari, Italy; 6 Biomedical Science Department, PhD School of Biomedical Science, University of Sassari, Sassari, Italy

**Keywords:** Maxillofacial surgery, Facial fractures, Epidemiology, Maxillofacial fractures, Traumatology, COVID-19, SARS-CoV-2

## Abstract

**Objective:**

The purpose of this study has been to analyze retrospectively the clinical records of patients admitted to a single Maxillofacial Surgery center in Northern Italy, evaluating the epidemiological differences during the different phases of the SARS-CoV-2 pandemic.

**Materials and Methods:**

The retrospective study included patients who had undergone surgery for facial bone fractures at the between February 23, 2020, and February 23, 2022. According to the date of admission, all the data were stratified into four chronological periods representing the four different phases of the SARS-CoV-2 pandemic in Italy in terms of restriction: pre-pandemic, first wave, partial restrictions and post-pandemic groups. Finally, any differences in epidemiological data between the groups were analyzed.

**Results:**

A total of 155 patients were included. The causes of admission of the pre-pandemic group demonstrated a statistically significant difference compared with the first wave group, whereas there were no differences between the pre-pandemic and the other two groups. The first wave group reported statistically significant differences in terms of the fracture etiology also with the partial restrictions and post-pandemic groups.

**Conclusions:**

During the COVID-19 pandemic, there were profound changes in the epidemiology of fractures influenced by the restrictive measures implemented by the government. At the end of the pandemic, the epidemiology of fractures returned to the same as in the pre-pandemic period.

## Introduction

Fractures of the facial bones are among the most frequent diagnoses on admission to maxillofacial surgery units. The epidemiology of this pathology is extremely variable and may be influenced by several factors such as life habits, sociocultural level and the general characteristics of the population [1].

On January 30, 2020, the World Health Organization declared a state of pandemic emergency due to the outbreak of the SARS-CoV-2 infection. In March 2020, as a result of the increase in infections, Italy adopted a series of restrictions on the movement of people and the closure of nonessential activities, a period commonly known as the lockdown. With the mitigation of the pandemic, a period of gradual reopening was therefore envisaged, which led to a resumption of normal daily life.

The profound change in people's lifestyles during the lockdown has had important effects on the epidemiology of maxillofacial fractures [2–7]. The overall number of cases significantly decreased and domestic accidents became the most frequent cause instead of interpersonal violence and road traffic accidents.

The few epidemiological studies published so far are subject to the limitation of analyzing only the differences in the frequency and etiology of facial fractures between the pre-pandemic period and the lockdown [2–6]. However, there are no studies that have analyzed the subsequent periods to understand whether, once the population has returned to a substantially normal lifestyle, differences continue to persist compared to the period before the pandemic. Studies of this type are fundamental to guide health systems in strategic choices and in the allocation of resources.

Therefore, the purpose of this study has been to analyze retrospectively the clinical records of patients admitted to a single Maxillofacial Surgery center in Northern Italy, evaluating the epidemiological differences during the different phases of the SARS-CoV-2 pandemic.

## Materials and Methods

This retrospective study included patients who had undergone surgery for facial bone fractures at the between February 23, 2020, and February 23, 2022. Patients not requiring surgical treatment were excluded from the study as complete clinical documentation would not be available.

According to the date of admission, all the cases were stratified into four chronological periods representing four different phases of the SARS-CoV-2 pandemic in terms of restrictions in Italy:

1. Pre-pandemic group: from June 23, 2019, to February 23, 2020.
2. First wave group: from February 23, 2020, to October 23, 2020, corresponding to the lockdown and immediate post-lockdown period.
3. Partial restrictions period group: from October 23, 2020, to June 23, 2021, corresponding to a period in which the Italian Ministry of Health had stratified the different areas of concern with different levels of restrictions (ranging from the closure of bars and clubs, social distancing, the obligation to wear a mask in closed places, smart working and the closure of leisure and sport clubs)
4. Post-pandemic group: from June 23, 2021, to February 23, 2022

For each individual included, data about gender, age, ethnic group, cause of admission and duration of hospital stay were collected. The facial fractures were classified as low and high energy based on their etiopathogenesis [8]. In the high impact group, we included all the injuries caused by a force 50 times or greater than the force of gravity (e.g., mandible, orbit and fronto-glabellar area injuries). In contrast, we classified as low impact all the fractures caused by forces less than 50 times the force of gravity such as zygomatic and nasal injuries.

The data were extracted from electronic clinical records by two independent researchers. The study was approved by the CEAVEC Ethical Committee Board (n.192-2022-OSS-AUSLBO).

All the data were independently reviewed and analyzed by the hospital statistician. The categorical variables are reported in numerals and percentages of the total. Descriptive statistics for the quantitative variables are given as the median [interquartile range (IQR)] or mean ± standard deviation (SD). The Fisher’s exact test was performed to evaluate the differences between the four groups in the categorical variables. A two-sided critical value of *p* < 0.05 was used to define the significance.

## Results

A total of 155 patients were included. Of these, 37 had been admitted for aggression and interpersonal violence, 43 for domestic accidents, 9 for sport accidents, 3 for suicide attempts, 54 for traffic collisions and 8 for work accidents. One had been admitted for reasons which were unclear.

**Fig. 1:**
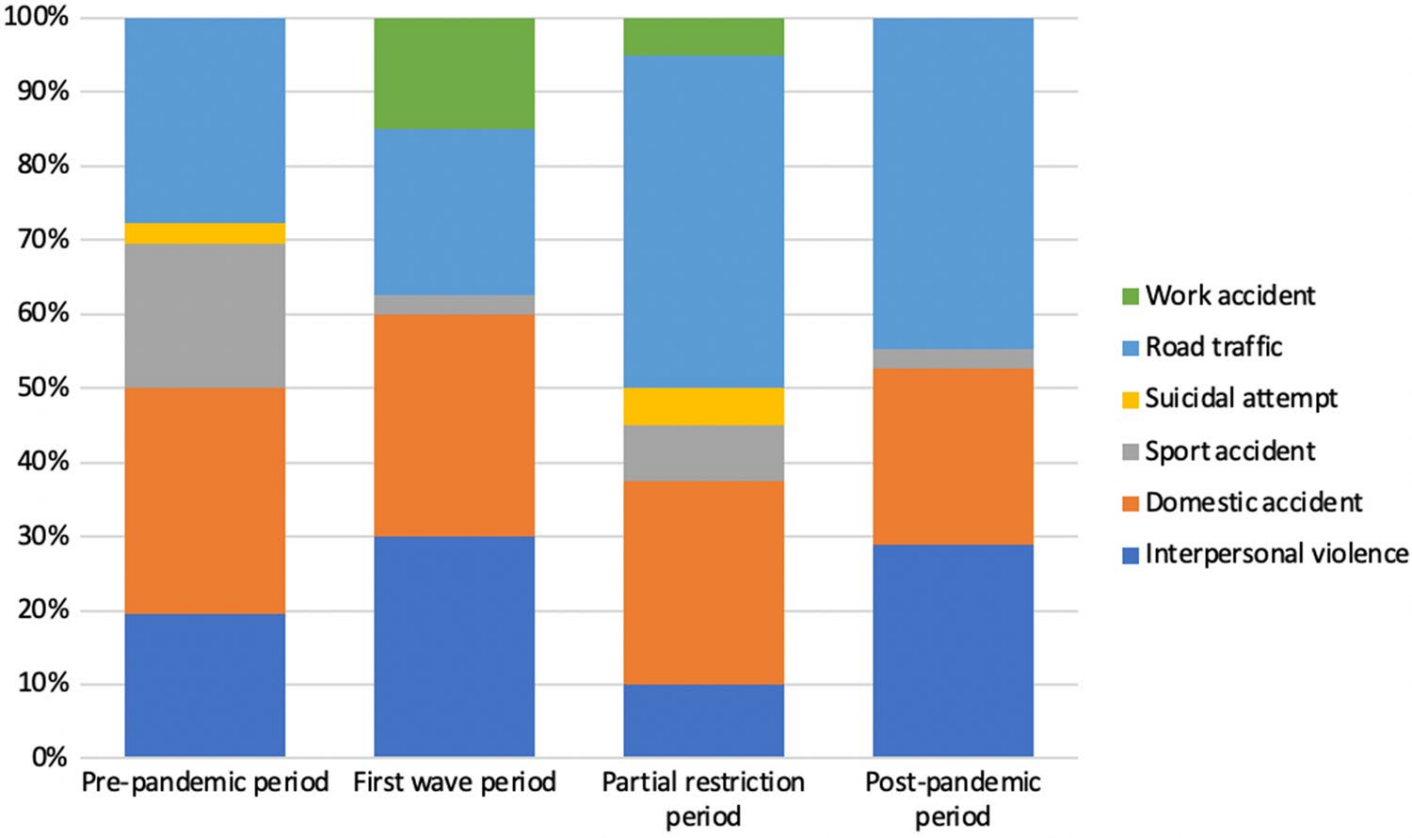
Causes of admission with reference to the different periods of the study

47 patients were females and 108 males. Among the individuals admitted to the Emergency Department for aggression, 97.3% were men. In contrast, women were more frequently involved in domestic accidents (53.5%). A statistically significant difference between the genders and the causes of admission was highlighted (*p* < 0.001) (Table 1). The patients included within the categories of interpersonal violence, aggression and sport accidents were younger than those admitted for other reasons (Table 1). All patients had undergone surgical treatment. Of these, only five had undergone closed treatment. In the pre-pandemic period, the most numerous group of the patients (30.6%) had been admitted for domestic accidents (Fig. 1), whereas, during the lockdown the two most numerous groups had been admitted for interpersonal violence (30%) and domestic accidents (30%). Road traffic accidents were the most frequent reasons for admission during both the partial restrictions (45%) and post-pandemic periods (44.7%) (Fig. 1).

**Table 1:** Descriptive analysis in relation to the causes of admission

Factor	Group	Interpersonal violence *N* (%)	Domestic accident *N* (%)	Sport accident *N* (%)	Suicidal attempt *N* (%)	Road traffic *N* (%)	Work accident *N* (%)	*p*-value
*N*		34	43	12	3	54	8	
Ethnic group	Asians	0 (0.0)	0 (0.0)	0 (0.0)	0 (0.0)	1 (1.9)	0 (0.0)	0.039
	Black	11 (32.4)	2 (4.7)	0 (0.0)	1 (33.3)	7 (13.0)	0 (0.0)	
	Caucasian	23 (67.6)	41 (95.3)	12 (100.0)	2 (66.7)	46 (85.2)	8 (100.0)	
Gender	Female	0 (0.0)	23 (53.5)	1 (8.3)	1 (33.3)	22 (40.7)	0 (0.0)	< 0.001
	Male	34 (100.0)	20 (46.5)	11 (91.7)	2 (66.7)	32 (59.3)	8 (100.0)	
Period	Pre-pandemic	7 (20.6)	11 (25.6)	7 (58.3)	1 (33.3)	10 (18.5)	0 (0.0)	0.004
	First wave	12 (35.3)	12 (27.9)	1 (8.3)	0 (0.0)	9 (16.7)	6 (75.0)	
	Partial restrictions	4 (11.8)	11 (25.6)	3 (25.0)	2 (66.7)	18 (33.3)	2 (25.0)	
	Post-pandemic	11 (32.4)	9 (20.9)	1 (8.3)	0 (0.0)	17 (31.5)	0 (0.0%	
Treatment	Closed	1 (2.9)	1 (2.3)	1 (8.3)	0 (0.0)	1 (1.9)	1 (12.5)	0.589
	Open	33 (97.1)	42 (97.7)	11 (91.7)	3 (100.0)	53 (98.1)	7 (87.5)	
Type of fracture	High energy	0 (0.0)	00 (0.0)	1 (8.3)	3 (100.0)	50 (92.6)	5 (62.5)	< 0.001
Age at admission median [IQR]		26.00 [23.00, 32.75]	58.00 [43.50, 73.00]	26.00 [19.75, 32.75]	61.00 [42.00, 72.00]	36.00 [26.00, 46.75]	44.00 [39.00, 57.50]	< 0.001
Discharge time median [IQR]		4.00 [3.00, 5.75]	5.00 [3.00, 8.00]	5.00 [3.75, 6.00]	15.00 [9.50, 29.00]	9.00 [6.00, 16.00]	5.50 [4.75, 11.75]	< 0.001

The Fisher’s exact test was then used to highlight differences in the causes of admission with respect to the different periods. In particular, the causes of admission of the pre-pandemic group were compared to each of the other groups (Table 2). The data demonstrate a statistically significant difference between the causes of admission between the pre-pandemic and the first wave groups, whereas there were no differences between the pre-pandemic group and the other two groups.

**Table 2:** Comparative analysis of the causes of admission between the pre-pandemic and the other groups

Etiology	Pre-pandemic *N* (%)	First wave *N* (%)	*p*-value
	37	40	
Interpersonal violence	7 (19.4)	12 (30.0%)	0.017
Domestic accident	11 (30.6)	12 (30.0%)	
Sport accident	7 (19.4)	1 (2.5%)	
Suicidal attempt	1 (2.8)	0 (0.0%)	
Road traffic accident	10 (27.8)	9 (22.5%)	
Work accident	0 (0.0)	6 (15.0%)	

Etiology	Pre-pandemic *N* (%)	Partial restrictions *N* (%)	*p*-value
	37	40	
Interpersonal violence	7 (19.4)	4 (10.0)	0.241
Domestic accident	11 (30.6)	11 (27.5)	
Sport accident	7 (19.4)	3 (7.5)	
Suicidal attempt	1 (2.8)	2 (5.0)	
Road traffic accident	10 (27.8)	18 (45.0)	
Work accident	0 (0.0)	2 (5.0)	

Group	Pre-pandemic *N* (%)	Post-pandemic *N* (%)	*p*-value
	37	38	
Interpersonal violence	7 (19.4)	11 (28.9)	0.062
Domestic accident	11 (30.6)	9 (23.7)	
Sport accident	7 (19.4)	1 (2.6)	
Suicidal attempt	1 (2.8)	0 (0.0)	
Road traffic accident	10 (27.8)	17 (44.7)	
Work accident	0 (0.0)	0 (0.0)	

The authors then compared the group of patients admitted during the first wave and those of the partial restrictions and post-pandemic periods, reporting statistically significant differences (Table 3).

**Table 3:** Comparative analysis of the causes of admission between the first wave and the partial restrictions and post-pandemic groups

Etiology	First wave *N* (%)	Partial restrictions *N* (%)	*p*-value
	40	40	
Interpersonal violence	12 (30.0)	4 (10.0)	0.027
Domestic accident	12 (30.0)	11 (27.5)	
Sport accident	1 (2.5)	3 (7.5)	
Suicidal attempt	0 (0.0)	2 (5.0)	
Road traffic accident	9 (22.5)	18 (45.0)	
Work accident	6 (15.0)	2 (5.0)	

Etiology	First wave *N* (%)	Post-pandemic *N* (%)	*p*-value
	40	38	
Interpersonal violence	7 (19.4)	11 (28.9)	0.040
Domestic accident	11 (30.6)	9 (23.7)	
Sport accident	7 (19.4)	1 (2.6)	
Suicidal attempt	1 (2.8)	0 (0.0)	
Road traffic accident	10 (27.8)	17 (44.7)	
Work accident	0 (0.0)	0 (0.0)	

## Discussion

Maxillofacial fractures constitute one of the most frequent pathologies treated at Maxillo-Facial Units. They represent an important public health and economic problem. The epidemiology is variable and dependent on socioeconomics factors, geographical area and cultural and racial differences [1]. The most common causes of facial fractures include assaults, falls, traffic accidents, sports accidents and work accidents [9].

National Healthcare Expenditure Accounts data in 2019 show that Italy spends about 118 billion euros per year on health care with the cost of maxillofacial injuries calculated to be approximately 160 million euros.

The incidence of maxillofacial fractures varies widely among different countries, and a large number of studies [10–12] have been performed regarding their epidemiology. The epidemiological analysis of maxillofacial fractures is essential to identify these injuries and to better address preventive measures and healthcare expenditure resources.

A common finding in many studies is that male patients are more frequently affected by maxillofacial bone trauma than female patients [13] probably due to a higher rate of mobility, an intense social interaction with a higher probability of being involved in criminal or violent situations and a greater inclination to participate in dangerous or extreme sports. This is consistent with our results; in our group, the majority of the patients included were males. In our experience, men were more frequently involved in interpersonal violence, whereas women in domestic accidents. In the literature, interpersonal violence has often been reported in patients living in socially disadvantaged conditions in the urban centers of developed countries [1, 14]. Therefore, the most common cause of facial trauma in males is road traffic accidents followed by assaults, falls, sports-related accidents and work-related accidents. The most frequent cause of facial injuries in females is road traffic accidents follow by accidental falls, assaults, work-related accidents and sport-related accidents.

As previously mentioned, our cohort reported a different epidemiology. This might be due to the peculiarity of the reference period. In fact, we report a greater frequency of traumas resulting from interpersonal violence as compared to road traffic collisions. The peculiarity of the period considered in the study, indeed, consists not only in the closure of commercial and recreational activities but also in the introduction of measures such as smart working.

As described in the literature [8], facial fractures, generally, tend to be mandibular fractures with a condylar involvement, whereas in interpersonal violence accidents, the zygomatic complex, orbit and nose are the most common bones involved. In this study, we have classified the fractures in terms of high and low impact. In agreement with the previously mentioned literature findings, in our cohort we found that the interpersonal violence injuries were predominantly nasal and zygomatic fractures (low impact fractures). In contrast to the scientific literature, we detected a lower frequency of high impact fractures (96 low impact and 59 high impact), a finding which might also be a result of the introduction of the lockdown restrictions.

The analysis of maxillofacial traumatic injuries revealed a predominance of young adult, male patients with the injuries most commonly caused by motor vehicle accidents. Our findings disagree with those presented in the literature in this case. Indeed, we report a median age at admittance for interpersonal violence of 26 years of age and for traffic road collisions of 36 years of age.

The severe acute respiratory syndrome SARS-CoV-2 was first recorded in China at the end of December 2019 and then spread like wildfire worldwide. Within a few weeks, it had had an immense impact on the population, community and individual behaviors and habits.

Since the start of the COVID-19 pandemic, maxillofacial elective surgery was gradually reduced nationwide and clinical work scaled down in order to keep services running without putting both the healthcare workers and patients at risk. As already shown elsewhere in the literature, national holidays, seasonal trends and natural disasters like the pandemic emergency influence the incidence of trauma [15].

The pandemic and the related restriction measures are linked to increases in depression and anxiety, isolation and loneliness, alcohol and cannabis misuse and unemployment and other economic problems [4, 16]. This may be the reason why in our analysis we have observed a prevalence of facial injuries mainly due to assaults and domestic accidents. In our cohort, we have also identified three cases of suicide attempts, two of which happened during the restriction period. Many experts have voiced concerns that mental, economic, behavioral and psychosocial problems linked to the COVID-19 pandemic might have contributed to a rise in suicidal behaviors [16].

Our statistical analysis has also highlighted that when the restrictions were stricter (the first wave period) an actual shift in the causes of admission for trauma was observed. We compared the pre-pandemic group causes of admission versus each single period afterward. Only the comparison with the first wave demonstrated a significant statistical difference (*p* = 0.002). This finding is confirmed by the further analysis performed by means of the Fisher’s exact test on the first wave group versus the restriction and post-pandemic groups. Furthermore, when looking at the descriptive analysis, it is striking that the admissions for traffic collisions were more frequent during the pre-pandemic (27.8%), partial restrictions (45%) and post-pandemic (44.7%) periods. In contrast, the majority of the patients admitted during the first wave lockdown were victims of interpersonal violence (30%) and domestic accidents (30%). In fact, during the first wave lockdown the rate of admissions for road traffic accidents was the lowest of all the four phases (22.5%), due to the introduction of government measures such as smart working and traveling admitted only for emergency reasons.

During the partial restrictions and post-pandemic periods, the rates for interpersonal violence and domestic accidents were 10, 28.9, 27.5 and 23.7%, respectively. These findings demonstrate that these rates were lower when the restrictions were loosened. This is probably a consequence of the fact that during the lockdown the forced house sharing and forced confinement at home may have generated a rise in aggression and an increase in domestic trauma.

It is interesting to note that, contrary to what might have been expected, the prevalence of maxillofacial traumas has not decreased during the lockdown period. This observation has already been reported in the literature by other authors who have not found significant differences in terms of the prevalence [2] and severity [4] of facial traumas during lockdown periods. This is due to a shift in the etiology of fractures, with a reduction in traumas due to road accidents and an increase in those related to other causes, such as interpersonal violence, domestic incidents and accidental falls.

The principal strength and novelty of this study lie in its pioneering analysis of the trends in the epidemiology of facial fractures throughout the various phases of the pandemic, extending into the post-pandemic period. This comprehensive approach allows for an assessment of whether the pandemic has had stable effects on people's habits sufficient to permanently alter the epidemiology of fractures. Understanding these changes is crucial, as it can inform future public health strategies and healthcare resource allocation. By identifying shifts in the causes and patterns of facial fractures, healthcare systems can better prepare for and respond to similar global health crises in the future, potentially leading to improved patient outcomes and more efficient use of medical resources.

However, the study is not without its limitations. Its retrospective nature is one such constraint, which may introduce biases related to data collection and analysis, limiting the ability to control for all potential confounding variables or to capture the full spectrum of relevant data. Additionally, being a single-center study poses challenges to the generalizability of the results. The data and conclusions drawn from one hospital or region may not accurately reflect the situation across the entirety of Italy, as regional variations in healthcare systems, population behaviors and pandemic impact can significantly influence the epidemiology of facial fractures.

## Conclusions

During the COVID-19 pandemic, there have been profound changes in the epidemiology of fractures influenced by the restrictive measures implemented by the government. At the end of the pandemic, the epidemiology of fractures resumed the same pattern as in the pre-pandemic period.
